# Photocatalytic generation of hydrogen by core-shell WO_3_/BiVO_4_ nanorods with ultimate water splitting efficiency

**DOI:** 10.1038/srep11141

**Published:** 2015-06-08

**Authors:** Yuriy Pihosh, Ivan Turkevych, Kazuma Mawatari, Jin Uemura, Yutaka Kazoe, Sonya Kosar, Kikuo Makita, Takeyoshi Sugaya, Takuya Matsui, Daisuke Fujita, Masahiro Tosa, Michio Kondo, Takehiko Kitamori

**Affiliations:** 1 Department of Applied Chemistry, School of Engineering, The University of Tokyo, 7-3-1 Hongo, Bunkyo, Tokyo 113-8656, Japan; 2 National Institute of Advanced Industrial Science and Technology (AIST), AIST Central 2-13, Tsukuba, Ibaraki 305-0047, Japan; 3 Chernivtsy National University, Institute of Physics, Engineering and Computer Science, Storozhynetska 101, Chernivtsy, 58000 Ukraine; 4 National Institute for Materials Science (NIMS), 1-2-1 Sengen, Tsukuba, Ibaraki 305-0047, Japan

## Abstract

Efficient photocatalytic water splitting requires effective generation, separation and transfer of photo-induced charge carriers that can hardly be achieved simultaneously in a single material. Here we show that the effectiveness of each process can be separately maximized in a nanostructured heterojunction with extremely thin absorber layer. We demonstrate this concept on WO_3_/BiVO_4_+CoPi core-shell nanostructured photoanode that achieves near theoretical water splitting efficiency. BiVO_4_ is characterized by a high recombination rate of photogenerated carriers that have much shorter diffusion length than the thickness required for sufficient light absorption. This issue can be resolved by the combination of BiVO_4_ with more conductive WO_3_ nanorods in a form of core-shell heterojunction, where the BiVO_4_ absorber layer is thinner than the carrier diffusion length while it’s optical thickness is reestablished by light trapping in high aspect ratio nanostructures. Our photoanode demonstrates ultimate water splitting photocurrent of 6.72 mA cm^−2^ under 1 sun illumination at 1.23 V_RHE_ that corresponds to ~90% of the theoretically possible value for BiVO_4_. We also demonstrate a self-biased operation of the photoanode in tandem with a double-junction GaAs/InGaAsP photovoltaic cell with stable water splitting photocurrent of 6.56 mA cm^−2^ that corresponds to the solar to hydrogen generation efficiency of 8.1%.

Bismuth vanadate (BiVO_4_) is one of the most promising materials for the photocatalytic production of hydrogen^1^ via water splitting with a relatively narrow bandgap of 2.4 eV in the monoclinic phase, excellent stability against photocorrosion and low cost. Theoretical solar to hydrogen (STH) efficiency of BiVO_4_ approaches 9.2% with the photocurrent of 7.5 mA cm^−2^ under the standard AM1.5G solar light illumination. Despite being a good absorber with a direct bandgap, BiVO_4_ has poor electron transport properties^2^ due to a high recombination rate of photogenerated carriers. As a result, BiVO_4_ is characterized by a short carrier diffusion length (*L*_*d*_) of around 70 nm^3^, which is the main reason why the first generation of BiVO_4_ photoanodes demonstrated small photocurrents of less than 1 mA cm^−2^ at 1.23 V_RHE_^4^,^5^,^6^,^7^,^8^,^9^.

In addition to poor electron transport properties, slow transfer of holes at the BiVO_4_/electrolyte interface is another performance limiting factor. Coupling of BiVO_4_ with RhO_2_^10^, cobalt-phosphate (CoPi)^11^,^12^,^13^,^14^ or FeOOH/NiOOH^15^ co-catalysts helped to improve the kinetics of oxygen evolution reaction (OER) significantly. Since the development of efficient OER co-catalysts, the bulk electronic conductivity was identified as a remaining performance bottleneck of BiVO_4_ photoanodes^3^. Although the attempts to enhance transport properties of BiVO_4_ by doping with Mo^14^ or W^12^,^13^ quickly raised photocurrent to 2.3 mA cm^−2^ 16, and then to 3.6 mA cm^−2^ 17, at 1.23 V_RHE_ by using a gradual doping profile and CoPi OER co-catalyst, these photocurrents were still smaller than 50% of the theoretically possible value of 7.5 mA cm^−2^. The combination of BiVO_4_ with more conductive WO_3_ in a form of a planar heterojunction resulted in similar photocurrents of 2.8 mA cm^−2^ for simple WO_3_/BiVO_4_ and 3.04 mA cm^−2^ for a WO_3_/SnO_2_/BiVO_4_ structure with a SnO_2_ blocking layer. The type II band alignment at the heterojunction interface helped to improve separation of the photogenerated carriers. However, the photocurrent remained limited, because the *L*_*d*_ was still shorter than the thickness of the BiVO_4_ film, that was required to gain a sufficient light absorption.

An alternative approach to compensate for the short *L*_*d*_ is to use an extremely thin absorber (ETA) heterojunction structure, where the BiVO_4_ absorber is thinner than the *L*_*d*_ while the optical thickness is reestablished by a structured interface with a high aspect ratio. The ETA structure significantly improves collection probability of photogenerated carriers, because they do not need to travel over large distances before separation. Another important advantage of the ETA structure is the efficient light scattering that increases the optical path through the device and thereby enhances the photon absorption. As a result, photocurrent in the photoanode can be maximized by separate optimization of optical and electronic thicknesses of the BiVO_4_ absorber.

The first WO_3_/BiVO_4_ heterojunction photoanode with a nanostructured interface was demonstrated by Su *et al.*^18^ Their photoanode was based on disordered WO_3_ nanowires (NW) prepared by a solvothermal method without formation of a fully developed ETA structure. As a result, the photocurrent was limited to less than 1 mA cm^−2^. Further progress was focused on preparation of fully developed and uniform 1D core-shell WO_3_/BiVO_4_ structures. Although the ETA photoanodes based on chemically prepared WO_3_-NWs demonstrated improved photocurrents of 2.4 mA cm^−2^ by Pilli *et al.*^19^ and 3.1 mA cm^−2^ by Rao *et al.*^20^, it was clear that the structurally defective WO_3_-NWs are responsible for high resistive losses in the structure. Thus, preparation of sufficiently conductive, uniform and vertically standing WO_3_ nanostructures with a high aspect ratio appeared as a key factor for realization of efficient WO_3_/BiVO_4_ heterojunction photoanodes.

In our previous work^21^ we firstly demonstrated a WO_3_/BiVO_4_ heterojunction photoanode based on WO_3_ nanorods (NRs) fabricated by Glancing Angle Deposition (GLAD). After application of the CoPi OER co-catalyst we achieved the photocurrent of 3.2 mA cm^−2^ at 1.23 V_RHE_, that was the record photocurrent among published for BiVO_4_ photoanodes at that time. We demonstrated that the WO_3_-NRs prepared by GLAD provide highly efficient pathways for photogenerated electrons and outlined that further optimization of the WO_3_-NRs/BiVO_4_ core-shell structure toward better conformality of the BiVO_4_ ETA layer should lead to a nearly theoretical photocurrent. Here we report the optimized WO_3_-NRs/BiVO_4_+CoPi photoanode with the record photocurrent of 6.72 mA cm^−2^ at 1.23 V_RHE_ that approaches 90% of the theoretically possible value. To the best of our knowledge, this is the highest photocurrent reported up to date for a water splitting photoanode.

During preparation of this manuscript Shi *et al.*^22^ reported a WO_3_-NRs/BiVO_4_:W,Mo-doped photoanode based on helical WO_3_-NRs deposited by GLAD. Their photoanode demonstrated the photocurrent of 4.2 mA cm^−2^ with CoPi and 5.35 mA cm^−2^ with FeOOH/NiOOH co-catalysts. Although their helical nanostructure showed complete absorption of the incident light, the absorption edge of the Mo,W-doped BiVO_4_ was blue-shifted due to doping. Also, the thickness of the BiVO_4_ layer in their nanostructure was rather small, that in turn required very long helical WO_3_ nanorods of 5.5 μm to gain the optical thickness. As a result, the combined effect of the blue shift and resistive losses in the long WO_3_ nanorods limited the photocurrent of their device to 5.35 mA cm^−2^, which is near 70% of the possible theoretical value. Also, Shi *et al.* attributed the performance enhancement to the helical morphology of their WO_3_-NRs. However, they did not compare the performance of helical and plain nanorods experimentally. Their conclusion about significant contribution of the helical morphology to the light trapping relies on the finite element frequency domain (FEFD) simulations of WO_3_-NRs without including the BiVO_4_ ETA layer into the model. From a ray-optics perspective, the absorption enhancement factor due to light trapping at rough interfaces is given by 4*n*^2^, where *n* is the refractive index of the absorber^23^. Since the refractive indexes of WO_3_ and BiVO_4_ are both close to 2.5^24^,^25^, we can expect sufficient light trapping even in case of plain nanorod morphology. Indeed, in this work we achieved 20% higher photocurrent than Shi *et al.* by using plain WO_3_-NRs of a much shorter length of only 2.5 μm and an undoped BiVO_4_+CoPi absorber layer with an optimized thickness of around 25 nm.

We also examined the combined influence of light intensity and temperature on the performance of our photoanode. Photocatalytic water splitting is a chemical reaction and thus will accelerate with increase of temperature according to the Arrhenius relation. Therefore, previously unused infrared light can heat the cell and contribute to water splitting by improving the reaction kinetics. It turns out that this feature is of special importance for the efficient performance of PEC under concentrated light, as it helps to significantly reduce recombination losses due to improved charge transfer kinetics at a photoanode/electrolyte interface and thus to avoid the typical sub-linear dependence of photocurrent on light intensity^26^. In this work we demonstrate that our photoanode achieves a stable photocurrent of 18.2 mA cm^−2^ under concentrated light of 3 suns at the cell temperature of 50 °C.

Spontaneous water splitting requires a photocatalytic material with conduction and valence bands positions that provide sufficient overpotentials for H_2_ and O_2_ evolution half-reactions. Unfortunately, the position of the BiVO_4_ conduction band does not fulfil that condition, and the photoanode needs an additional bias potential to drive the H_2_ evolution half-reaction. Construction of a photoanode/photovoltaic tandem is a good approach to fabricating a self-biased water splitting cell. Previously, mechanically stacked tandems, based on a dye-sensitized solar cell (DSSC) with Fe_2_O_3_ or WO_3_ photoanodes^27^, and monolithic tandems, based on single- or double-junction a-Si solar cells with BiVO_4_:Mo+CoPi photoanode layers^17^, demonstrated self-biased photocurrents of 1.34, 2.23, 3.0 and 4.0 mA cm^−2^, respectively. In all the cases the performance was affected by inferior transparency of the top BiVO_4_ photoanode. Since our photoanode can efficiently operate under concentrated light, which saves a lot of lateral space, we can accommodate alternative tandem configurations. Here we demonstrate a PEC-PV tandem device based on a WO_3_-NRs/BiVO_4_+CoPi photoanode biased by a GaAs/InGaAsP solar cell that operates under reflected light from the photoanode. The PEC-PV tandem achieves a stable water splitting photocurrent of 6.56 mA cm^−2^ at standard conditions and 18.17 mA cm^−2^ at 3 suns and elevated cell temperature of 50 °C.

## Results and Discussion

**Figure 1:**
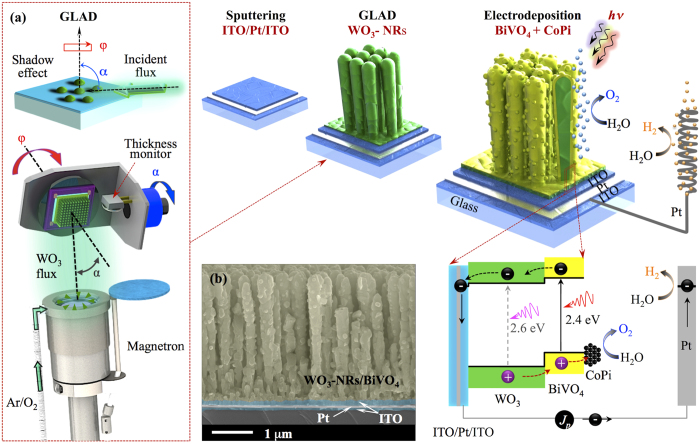
Schematic illustration of a core-shell WO_3_-NRs/BiVO_4_ photoanode fabricated by glancing angle deposition (GLAD) of WO_3_-NRs followed by electrodeposition of BiVO_4_+CoPi. The inset (**a**) illustrates GLAD. The SEM image (**b**) shows a cross section of the ITO/Pt/ITO/WO_3_-NRs/BiVO_4_+CoPi photoanode.

We fabricated the photoanode by a combination of GLAD of WO_3_-NRs and subsequent Electrochemical Deposition (ED) of BiVO_4_ and CoPi (Fig.1). The details of the fabrication process can be found in experimental section. Scanning electron microscopy (SEM) studies of the optimized WO_3_-NRs and WO_3_-NRs/BiVO_4_ reveals that the nanorods are well separated and uniform with the length of 2.5 μm and the average diameter varying from 200 to 300 nm along the nanorods (Supplementary Fig. S1). This length was selected as optimal based on our previous work^21^. In order to optimize the thickness of the electrodeposited BiVO_4_ layer we studied the dependence of the photocurrent of WO_3_-NRs/BiVO_4_ samples measured at 1.23 V_RHE_ under the standard AM1.5G illumination on the total charge density that passed during the electrodeposition of BiVO_4_. The total charge density is proportional to the quantity of the deposited BiVO_4_ and thus to the average thickness of the BiVO_4_ layer. The increase of the BiVO_4_ layer thickness boosts light absorption and thus the photocurrent. When the thickness of the BiVO_4_ becomes comparable with the *L*_*d*_, the bulk recombination starts to prevail and the photocurrent decreases. The SEM observation of the samples reveals that the electrodeposition starts from the conformal growth of the BiVO_4_ layer and then proceeds with formation of hemispherical clusters. Finally, the overgrown clusters fill the gaps between the nanorods (Supplementary Fig. S2). The Scanning Probe Microscopy (SPM) studies of the topography and local current maps of WO_3_-NRs and WO_3_-NRs/BiVO_4_ samples indicate that the WO_3_-NRs are coated by a conformal BiVO_4_ layer with hemispherical clusters, that corresponds to the “layer-plus-island” Stranski–Krastanov growth mode during the electrodeposition (Supplementary Fig. S3). Also, the elemental distributions of W, Bi and V measured across a single WO_3_ nanorod with a BiVO_4_ layer by energy dispersive X-ray spectroscopy confirms its core-shell structure with Bi and V maxima near the edge of the nanorod (Supplementary Fig. S4). The monoclinic phases for WO_3_^28^ nanorods with triplet peaks at 23.0° (002), 23.5° (020) and 24.3° (200) and BiVO_4_^29^ with characteristic peaks at 18.9° (110), 29.2° (112) and 31.0° (200) were confirmed by XRD analysis (Supplementary Fig. S5).

The subsequent photo-assisted electrodeposition of a CoPi OER co-catalyst elevated the photocurrent density from 5.45 mA cm^−2^ to 6.72 mA cm^−2^ at 1.23 V_RHE_ for the optimized sample (Supplementary Fig. S2a). Figure S6 shows I-V characteristics of optimized WO_3_-NRs, WO_3_-NRs/BiVO_4_ and WO_3_-NRs/BiVO_4_+CoPi photoanodes measured in a three-electrode configuration under standard conditions. According to the commonly accepted PEC characterization protocol^30^, developed to standardize evaluation of water splitting PEC devices, the photoanode performance has to be characterized in a two-electrode configuration, because application of a potential bias versus a reference electrode excludes the second half-reaction at a counter electrode. For that reason, we performed further PEC characterizations in the two-electrode configuration with a Pt counter electrode. After comparing the I-V characteristics of the same photoanode measured in two- and three-electrode configurations, we estimated that the same photocurrent of 6.72 mA cm^−2^ measured at 1.23 V_RHE_ in the three-electrode configuration is achieved at the bias of 1.02 V in the two-electrode configuration (Supplementary Fig. S7). Since the onset of the dark current occurs only at 1.2 V, we selected 1 V as a standard bias to measure incident photon-to-current efficiency (IPCE), photocurrent-time (*J*_*p*_*-t*) stability profiles and direct O_2_/H_2_ evolution rates in the two-electrode configuration. As will be shown later, the I-V characteristic of the solar cell, that we used to construct the self-biased water splitting tandem device, also intersects the I-V characteristic of our photoanode at around 1 V and thus seamlessly substitutes the external bias.

**Figure 2:**
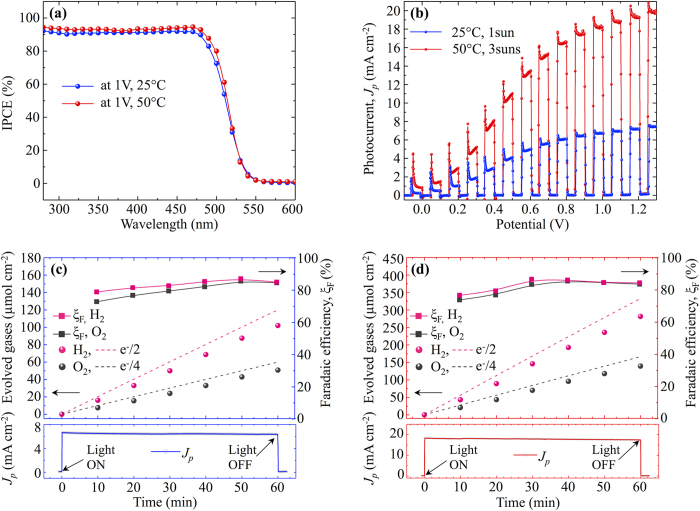
Photoelectrochemical performance of a WO_3_-NRs/BiVO_4_+CoPi photoanode measured by the two-electrode method under the bias of 1 V. (**a**) IPCE measured at 25 °C (blue) and 50 °C (red), (**b**) I-V characteristics measured under chopped light at 1 sun, 25 °C and at 3 suns, 50 °C. (c) and (d) gas production rates (circles), faradaic efficiencies (rectangles) and theoretical gas production rates (dashed lines) of O_2_ (black) and H_2_ (red) for 1sun, 25 °C (**c**) and for 3 suns, 50 °C (**d**) with simultaneously recorded *J*_*p*_*-t* profiles.

Figure 2 shows IPCE, chopped light I-V characteristics, O_2_/H_2_ evolution rates accompanied with faradaic efficiencies and *J*_*p*_*-t* stability profiles measured under standard conditions (1 sun, 25 °C) and under the combination of concentrated light and elevated temperature (3 suns, 50 °C). The IPCE characteristics measured at 25 °C and 50 °C show similar dependence on wavelength with the optical band onsets at 516 nm (2.4 eV) and plateaus in shorter wavelengths that reach 90% and 92%, respectively. The theoretical photocurrent of 6.73 mA cm^−2^ obtained by integrating the product of the IPCE measured at 25 °C with the AM1.5G photon flux over all wavelengths is close to the experimental value of 6.72 mA cm^−2^ at the bias of 1 V. Similar calculations for the IPCE measured at 50 °C and 3×AM1.5G photon flux give the photocurrent value of 20.9 mA, which is higher than the experimental value of 18.2 mA cm^−2^ implying increased recombination at higher light intensities. Figure 2c,d show the gas production rates of O_2_ and H_2_ with simultaneously recorded *J*_*p*_*-t* profiles. The H_2_ and O_2_ evolved at stoichiometric ratio with the H_2_ generation rates of 102 μmol h^−1^ cm^−2^ (at 1 sun, 25 °C) and 281 μmol h^−1^ cm^−2^ (at 3 suns, 50 °C). The *J*_*p*_*-t* curves were used to calculate the theoretical gas production rates and the faradaic efficiencies. The faradaic efficiencies reach 80% within the first 15 minutes and later saturate at ~85%, which is a typical value for PEC reactors with a single compartment where oxygen dissolved from the photoanode can undergo a partial back reaction at the Pt counter electrode.

The systematic studies of the photonode performance under different light intensities (0.5-3 suns) and cell temperatures (25–50 °C) reveal non-linear dependence of the photocurrent on the light flux (see Supplementary Fig. S8). The combined effect of light intensity (*I*) and temperature on the photocatalytic performance of BiVO_4_ has not been studied before. According to Tabata *et al.*^26^, the hydrogen evolution rate for photocatalytic K_4_Nb_6_O_17_ nanoparticles is proportional to *I*^0.92^ at low light intensities (<1 sun) and to *I*^0.52^ at high light intensities. A similar non-linear response to the light intensity was reported in other studies of photocatalytic water splitting^31^,^32^ and organic pollutants degradation^33^,^34^ by TiO_2_. The linear dependence of the photocurrent on the photon flux is valid as long as the photocatalytic reaction at the photoanode/electrolyte junction is faster than recombination rate. As the light intensity increases, the recombination rate becomes dominant causing a half-order dependence on light intensity. Since the sub-linear response represents competition between photocatalytic reaction and recombination rates, the former can be improved by increasing the cell temperature according to the Arrhenius kinetics law. The concept of positive influence of elevated temperature on photocatalytic water splitting was firstly proposed by Licht^35^ and experimentally reported by Hong *et al.*^36^ for TiO_2_ and by Katakis *et al.*^37^ for WO_3_. Here we used this approach to improve the photocatalytic reaction kinetics at the BiVO_4_+CoPi/electrolyte interface and extend the linear dependence of the photocurrent on the photon flux to higher light intensities.

The photocurrent (*J*_*p*_) of the optimized WO_3_-NRs/BiVO_4_+CoPi photoanode measured under 1 V bias *vs* a Pt counter electrode shows a typical sub-linear dependence on light intensity at room temperature with *J*_*p*_ ~ *I*^*m*^ and *m* = 0.54 (Supplementary Fig. S8a). This behavior corresponds to the domination of recombination at high light intensities. The intensity exponent *m* then increases toward unity with increasing temperature and reaches 0.93 at 50 °C due to improved reaction kinetics (Supplementary Fig. S8b). It is important to mention that solar thermal collectors with anti-reflecting flat panels can reach temperatures of around 50–60 °C even under 1 sun illumination^38^, while concentrated sunlight can easily heat a water splitting cell to 50–60 °C without special measures (Supplementary Fig. S9). The photocurrent of 18.2 mA measured at 3 suns and 50 °C corresponds to the quantum efficiency of 81%. As far as we know, this is the first report of efficient water splitting by a WO_3_-NRs/BiVO_4_ photoanode under concentrated light.

The water splitting photocurrent can be described as *J*_*p*_* = J*_*A*_* × P*_*sep*_* × P*_*inj*_, where *P*_*sep*_ and *P*_*inj*_ are separation and injection efficiencies that represent the fractions of holes that reach the photoanode/electrolyte interface and injected into the electrolyte to oxidize water, respectively, while *J*_*A*_ is the rate of photon absorption expressed as a current density. The separation and injection efficiencies characterize bulk and surface recombination processes. Dotan *et al.*^39^ proposed a method to independently estimate *P*_*sep*_ *and P*_*inj*_ by measuring *J*_*p*_ in the electrolyte that contains hole scavenger such as H_2_O_2_. This method relies on the assumption that the hole scavenger (H_2_O_2_) removes the injection barrier making *P*_*inj*_ = 1 without affecting the charge separation. We characterized our photoanodes in the electrolyte containing 0.5 M of H_2_O_2_ under different light intensities and found no dependence on temperature or significant difference between samples with and without CoPi (see Supplementary Fig. S8c) in the high potential region around 1 V. According to Dotan *et al.*^39^ the independent estimation of *P*_*sep*_ and *P*_*inj*_ can be done only in the case of absence of current transients in H_2_O_2_ containing electrolyte under chopped light. The positive current transients upon turning the light on correspond to the accumulation of holes at the electrode/electrolyte interface. In our case we always observe current transients in the low voltage region (see Supplementary Fig. S14). The transient spikes diminish significantly in the high voltage region and upon temperature increase. This means that independent estimation of *P*_*sep*_ and *P*_*inj*_ is partially possible only in the high potential region. Supplementary Fig. S8d shows *P*_*sep*_ and *P*_*inj*_ *vs* light intensity for two different temperatures 25 °C and 50 °C. The *P*_*sep*_ is almost constant around 90–95% and does not depend on light intensity and temperature. This is expected behavior for the core-shell ETA structure, where the diffusion length of the photogenerated carriers is much longer than the thickness of the absorber layer. Indeed the diffusion length in BiVO_4_ is 80 nm while the thickness of the BiVO_4_ layer in our core-shell WO_3_-NRs/BiVO_4_ nanostructure is only 25 nm. As a result, *P*_*sep*_ is usually close to 1, because electrons can quickly reach the heterojunction interface. In contrast *P*_*inj*_ strongly varies with the light intensity and temperature due to competing recombination at surface traps and photocatalytic reaction at the photoanode/electrolyte junction governed by Arrhenius kinetics law.

**Figure 3:**
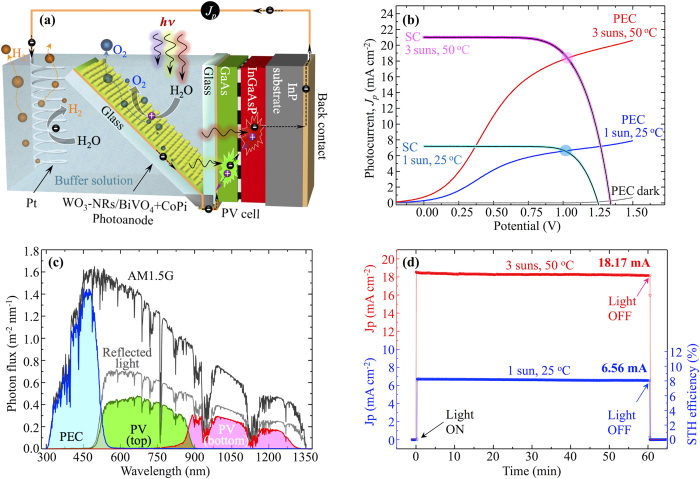
Characterization of PEC-PV tandem device. (**a**) Schematic illustration of the PEC-PV tandem with the PV cell operating under reflected light from the photoanode. (**b**) I-V characteristics of the PV cell and the photoanode measured at standard (1 sun, 25 °C) and concentrated light (3 suns, 50 °C) conditions. (**c**) Utilization of the incident AM1.5G solar light by the tandem device calculated from the IPCE of the PEC-PV tandem sub-cells and the reflectance spectra of the photoanode. (**d**) *J*_*p*_*-t* profiles measured for the PEC-PV tandem at 1 sun, 25 °C (blue) and 3 suns, 50 °C (red).

We demonstrated self-biased water splitting by assembling a tandem device where the WO_3_-NRs/BiVO_4_+CoPi photoanode is biased by a double-junction mechanically stacked GaAs/AlGaAsP photovoltaic cell. The PV cell was prepared following the previous work of Makita *et al.*^40^ with the top and the bottom cells bonded together by aligned metal nanoparticle arrays. The details on the construction and characterization of the PEC-PV tandem cell can be found in experimental section (also see Supplementary Figures S10 and S11). In order to avoid optical losses associated with inferior transparency of the nanostructured WO_3_-NRs/BiVO_4_ photoanode, we located the PV cell parallel to the incident light in such a way that it operated under the light reflected from the photoanode Fig. 3a). The incorporation of the ITO/Pt/ITO stack allowed us to simultaneously maximize reflectance and minimize resistive losses in the photoanode. We would like to emphasize that this configuration can be easily used in a water splitting panel module with segmented light concentrators, such as the one proposed by Turner^41^. Figure S12 shows a possible structure of the module that accommodates O_2_ and H_2_ collecting tranches fitted with the PEC-PV tandem and the Pt counter electrode, respectively, and with hemispherical light concentrators on the top of the module. Other types of optical concentrators that are already developed in the field of low concentration photovoltaics (low-CPV) can also be used to enable non-tracking operation^42^.

Figure 3b shows separately measured I-V characteristics of the PV and the PEC cells at 1 sun (25 °C) and 3 suns (50 °C) with intersection points at 6.6 mA (at 1.01 V) and 18.3 mA (at 1.02 V), respectively. Although our photoanode is located at 45° with respect to the incident light, its I-V characteristic does not differ much from the one measured at the normal incident angle. This benefit arises from the long core-shell WO_3_/BiVO_4_ nanorods that can capture light equally efficiently from any incident angle in contrast to photoanodes based on flat films. Figure S13 compares I-V characteristics measured at 90° and 45° incident angle for the photoanodes based on core-shell nanorods and flat films. The flat film photoanode shows notable difference in the photocurrent depending on the incident angle, which is mainly attributed to reflective optical losses typical for flat films.

The I-V characteristics of the PV cell were measured under the light reflected from the photoanode. We also measured the I-V characteristics of the PV cell without the photoanode in the tandem assembly to confirm that the PV cell is oriented parallel to the incident light and does not receive additional light from parasitic reflections (see Supplementary Fig. S10c). Indeed, the I-V characteristics in the absence of the photoanode were very close to the ones measured at dark conditions. Figure 3d shows the *J*_*p*_*-t* profiles measured for the tandem cell at 1 sun, (25 °C) and at 3 suns (50 °C) with stabilized photocurrents of 6.56 mA and 18.17 mA, respectively. The photocurrent of 6.56 mA corresponds to the theoretical STH efficiency of 8.1%, which is 1.65 times higher than the previous record of 4.92% reported for BiVO_4_:W,Mo/double-junction a-Si tandem. The photocurrent of 18.17 mA at 3 suns corresponds to the STH efficiency of 7.5%. Figure 3c shows utilization of the incident AM1.5G solar light by the tandem device that is calculated from the IPCE spectra of the PEC-PV tandem subcells and the reflectance spectra of the photoanode. The IPCE spectra of the PV cell and the PEC-PV tandem are shown in Supplementary Figures S10a and S10b, respectively. The reflectance of the photoanode was not exceeding 60% in the wavelength region >516 nm due to highly nanostructured ETA configuration. This is the reason why we had to use a highly efficient GaAs/AlGaAsP PV cell in order to generate the matching photocurrent under the weak reflected light. Although, operations under concentrated light can compensate the high cost of the PV cell to some degree, we acknowledge that a more economically viable solution has to be used in the future. Previously, PV assisted photoelectrolysis based on a GaAs/GaInP_2_ double-junction solar cell^43^ or CIGS mini-modules^44^ demonstrated STH efficiencies of 16% and 10.5%, respectively. However, in both cases a highly acidic 3 M H_2_SO_4_ electrolyte was used to achieve efficient photoelectrolysis, which raises concerns about stability of the cells. Also, PV cells with rather high open-circuit potential V_oc_ > 1.6 V are required to provide sufficient overpotential for the photoelectrolysis. In contrast, our photoanode efficiently works in a neutral electrolyte at pH = 7 and requires a much lower bias of around 1 V, that is in the range of recently developed inexpensive perovskite solar cells with V_oc_ of 1.15 V^45^. Furthermore, high optical losses originating from low reflectance of the photoanode at λ > 516 nm can be avoided by using a spectral splitting dichroic mirror that provides dedicated portions of the solar spectrum to the PV cell and to the photoanode separately. Therefore, we believe that an economically viable PEC-PV tandem based on a WO_3_-NRs/BiVO_4_ photoanode with STH of around 8% can be realized in the near future.

In conclusion, we utilized an extremely thin absorber concept to fabricate a highly efficient water splitting photoanode based on a WO_3_-NRs/BiVO_4_+CoPi core-shell nanostructured heterojunction with the record photocurrent of 6.72 mA measured at 1.23 V_RHE_ under the AM1.5 simulated solar light. To the extent of our knowledge, this is the highest photocurrent reported up to the date among all photoanode materials. Also, we firstly demonstrated an efficient performance of a WO_3_-NRs/BiVO_4_-based photoanode under concentrated light. We found that an enhanced recombination in BiVO_4_, that leads to sub-linear dependence of the photocurrent on the photon flux, can be compensated by elevating operation temperature of the cell, which significantly improves photocatalytic reaction kinetics at the BiVO_4_+CoPi/electrolyte interface. As a result, we were able to extend the near linear dependence of the photocurrent on the photon flux to higher light intensities and demonstrate a stable photocurrent of 18.2 mA measured at 3 suns and 50 °C under the equivalent bias of 1 V. Finally, we constructed a tandem device based on a WO_3_-NRs/BiVO_4_+CoPi photoanode and a GaAs/InGaAsP solar cell operating under reflected light from the photoanode with a stable water splitting photocurrent of 6.56 mA corresponding to the STH efficiency of 8.1%.

## Methods

### Fabrication of WO_3_-NRs/BiVO_4_+CoPi heterojunction photoanodes

Depositions of a ITO/Pt/ITO (150 nm/50 nm/150 nm) stack film and 2.5 μm long WO_3_-NRs were performed in a multi-magnetron glancing angle deposition (GLAD) system with a 3D rotation stage that allows positioning of the substrate over each magnetron at a desired distance and normal or glancing angle without breaking the vacuum. The formation of uniform WO_3_ nanorods instead of a compact thin film was achieved by deposition at a glancing angle, where the shadowing effect^46^ prevents deposition of incident atoms behind spontaneously formed islands (see Fig. 1a). As a result, the morphology of the growing film breaks to columnar while a continuous substrate rotation directs formation of vertically standing and separated nanorods. The as-grown amorphous WO_3_-NRs were converted to a crystalline monoclinic phase by annealing in air at 575 °C for 4.5 hours. The BiVO_4_ conformal layer was deposited over the WO_3_-NR_S_ by a modified electrodeposition method proposed by Seabold *et al.*^47^ from the electrolyte prepared by dissolution of 10 mM Bi(NO_3_)_3_ in 35 mM VOSO_4_ (adjusted to pH = 0.5 by HNO_3_) and then adjusted to pH = 4.7 with a 2 M sodium acetate solution and a few drops of HNO_3_. The electrodeposition was conducted in a two electrode configuration at 55 °C under the constant potential of 0.21 V *vs* a Pt counter electrode. The photoanodes were then annealed in air at 500 °C for 2 hours to convert the amorphous BiVO_4_ layer to a crystalline monoclinic phase. The CoPi OER co-catalyst was deposited by the photo-assisted electro-deposition (PED) method following the recipe published by Li *et al.*^3^,^6^ from the solution of 0.15 M cobalt nitrate in 0.1 M potassium phosphate buffer at constant photocurrent of ~10 μA cm^−2^ under 1 sun AM1.5G illumination during 500 s. We also prepared photoanode based on flat film WO_3_/BiVO_4_ heterojunction with 900 nm thick compact WO_3_ layer and 60 nm thick BiVO_4_ layer by using the same fabrication procedure, but without the GLAD regime.

### Characterization methods

The photoelectrochemical (PEC) characterizations of the photoanodes were conducted according to the standard PEC characterization protocol^7^ in potassium phosphate buffer solution (pH = 7) by a standard three-electrode method with Ag/AgCl reference and Pt counter electrodes, and by a two electrode method with the bias applied *vs* a Pt counter electrode. The simulated AM1.5G solar light was adjusted by using an NREL calibrated photodetector. The evolution rates of oxygen and hydrogen were directly measured in an airtight PEC cell connected to a gas micro-chromatograph. The WO_3_-NRs/BiVO_4_ core-shell nanostructures were characterized by X-Ray Diffraction (XRD), Scanning Electron Microscopy (SEM), Energy Dispersive X-ray Spectroscopy (EDS) and Scanning Probe Microscopy (SPM).

### Construction of a PEC-PV tandem device

The PV cell consisted of two mechanically stacked GaAs and AlGaAsP solar cells. The p-n junction layers of GaAs (Eg = 1.42 eV) and In_0.775_Ga_0.225_As_0.489_P_0.511_ (Eg = 1.0 eV) were fabricated on GaAs and InP substrates, respectively, by solid source MBE (ss-MBE). Then the GaAs epitaxial layer was separated from the substrate by the epitaxial lift-off (ELO) technique and connected to the AlGaAsP bottom cell through aligned Pd nanoparticle arrays as shown on Fig. S10. The structure was finalized by fabrication of the top AuGe/Ni/Au metal grid and bottom Ti/Au contacts. For more details on the growth of the GaAs and InGaAsP p-n junction layers by ss-MBE, and the transfer of the GaAs layers by ELO and interconnection of the layers by self-assembled Pd nanoparticle arrays, refer to previous works of Makita *et al.*^40^,^48^, Sugaya *et al.*^49^,^50^ and Mizuno *et al.*^51^ The solar cell was encapsulated between two thin glass plates with a UV-cured epoxy seal. The PEC-PV tandem was assembled by using a V-shape support where the PEC and the PV cells were located at 45° and parallel to the incident light, respectively. The size of the solar cell was 4 × 4 mm^2^. The widths of the photoanode and the PV cell were 4 mm while the length of the photoanode was equal to the width multiplied by 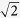, i.e. 5.65 mm. Since the photoanode was located at 45° to the incident light, the illuminated area of the tandem device was equal to 4 × 4 mm^2^. In that configuration the PV cell operated only under the light reflected from the photoanode. The I-V characteristics of the solar cell measured under normal illumination and under the light reflected from the photoanode are shown in Fig. S10c and confirm that the PV cell in the PEC-PV tandem was indeed oriented parallel to the incident light and did not receive additional light from parasitic reflections.

## Additional Information

**How to cite this article**: Pihosh, Y. *et al.* Photocatalytic generation of hydrogen by core-shell WO_3_/BiVO_4_ nanorods with ultimate water splitting efficiency. *Sci. Rep.* **5**, 11141; doi: 10.1038/srep11141 (2015).

## Supplementary Material

Supplementary Information
