# Supplementary material for: Photocatalytic generation of hydrogen by core-shell WO3/BiVO4 nanorods with ultimate water splitting efficiency
Source: Sci Rep. 2015 Jun 8;5:11141. doi: 10.1038/srep11141 (PMC4459147; doi:10.1038/srep11141)
Supplement: Supplementary Information [file srep11141-s1.pdf]

Supplementary Information

Photocatalytic generation of hydrogen by core-shell $WO_3/BiVO_4$ nanorods with ultimate water splitting efficiency

Yuriy Pihosh,[1, §, *] Ivan Turkevych,[2, 4, §] Kazuma Mawatari,[1] Jin Uemura,[1] Yutaka Kazoe,[1] Sonya Kosar,[1, 3] Kikuo Makita,[2] Takeyoshi Sugaya,[2] Takuya Matsui,[2] Daisuke Fujita,[4] Masahiro Tosa,[4] Michio Kondo,[2] and Takehiko Kitamori[1,*]

[1]*Department of Applied Chemistry, School of Engineering, The University of Tokyo, 7-3-1 Hongo, Bunkyo, Tokyo 113-8656, Japan*

[2]*National Institute of Advanced Industrial Science and Technology (AIST), AIST Central 2-13, Tsukuba, Ibaraki 305-0047, Japan*

[3]*Chernivtsy National University, Institute of Physics, Engineering and Computer Science, Storozhynetska 101, Chernivtsy, 58000 Ukraine*

[4]*National Institute for Materials Science (NIMS), 1-2-1 Sengen, Tsukuba, Ibaraki 305-0047, Japan*

---

[*] *Corresponding authors email (kitamori@icl.t.u-tokyo.ac.jp; pihosh@icl.t.u-tokyo.ac.jp)*

[§] *These authors contributed equally.*

Fabrication of WO_3-NRs/$BiVO_4$+CoPi heterojunction photoanodes. At first, we deposited a compact ITO film with the thickness of about 150 nm on fused silica substrates (5x5cm) at normal incidence angle (α=0º) from the ITO target (99.999%, Furuchi Chem. Co.) in the Ar:O_2 (15:0.3 SCCM) mixture and working pressure of 0.6 Pa. Then, the stage was turned toward the Pt target (99.999%, Furuchi Chem. Co.) to deposit a thin Pt film (~50 nm) in Ar atmosphere at the working pressure of 2 Pa. In the next step, the Pt film was encapsulated by the deposition of the second ITO layer (150 nm), as described above. The ITO/Pt/ITO stack has a low sheet resistance of ~3-4 Ω/□ due to the encapsulated Pt layer, which simultaneously acts as a back reflector. Since the Pt layer was encapsulated and had no contact with the electrolyte, it did not participate in the electrochemical reaction and thus in future could be substituted by a less expensive metal, such as Ag or Al. After the deposition of the ITO/Pt/ITO stack we set the sample over the third magnetron with W target (99.99%, Advantec Co.) and changed the stage position to the GLAD regime with α=85º to the substrate normal. The WO_3-NRs were deposited in the GLAD regime by reactive sputtering in the O_2:Ar (9.6 : 11 SCCM) mixture and low working pressure of 0.3 Pa with the constant speed of substrate rotation of 45 rpm. The fabrication of WO_3-NRs was finalized by annealing in air at 575 °C for 4.5 h.

The precursor solution for the electrodepositon of $BiVO_4$ was prepared by dissolving 10 mM of $Bi(NO_3)_3$ in a solution of 35 mM $VOSO_4$ adjusted to pH = 0.5 with HNO_3. The Bi(III) is soluble at pH < 2, however no film can be formed in such acidic solution. Therefore, at first the pH of the electrolyte was raised to 5.1 by 2 M sodium acetate solution and then stabilized at pH = 4.7 by adding a few drops of concentrated HNO_3, since V(IV) starts to precipitate at pH > 5.

The electrodeposition of $BiVO_4$ was conducted at potentiostatic conditions in the two electrode configuration with the bias of 0.21 V applied between ITO/Pt/ITO/WO_3-NRs as a working electrode and a Pt mesh as a counter electrode. The deposition of amorphous $BiVO_4$ was carried out at 55 °C by varying the deposition time from 35 to 270 s. All freshly prepared samples were rinsed with distilled water, dried in the N_2 steam and then annealed in air at 500 °C for 2 hours to convert the amorphous layer into a crystalline monoclinic $BiVO_4$.

The Co-Pi OER co-catalyst was deposited on the surface of $BiVO_4$ from a solution of 0.15 M cobalt nitrate in 0.1 M potassium phosphate buffer by a photo-assisted electrodeposition under 1 sun AM1.5G illumination. The sample was biased *vs* a counter Pt mesh electrode at galvanostatic conditions to keep the photocurrent at ~10 $\mu A\ cm^{-2}$. The optimized deposition time was found to be 500 s. The resulting photoanodes were rinsed with distilled water and dried under a gentle N_2 flow. The photoanodes based on flat film WO_3/$BiVO_4$ heterojunction were prepared by using the same fabrication procedure, but without the GLAD regime. All chemicals were purchased from Wako.

The photoelectrochemical (PEC) characterizations of the photoanodes were conducted according to the standard PEC characterization protocol in a potassium phosphate buffer solution (pH=7) by using ALS/CHI (608D) potentiostat and a standard three-electrode method with a Pt counter electrode and an Ag/AgCl

reference electrode. Two electrode measurements were conducted by following the same protocol. The I-V characteristics and the photocurrent-time (J_p-t) profiles were recorded under simulated solar light provided by a solar simulator (PEC-L01, Peccel Co.). The light intensity was adjusted by using an NREL calibrated photodetector.

The V_{RHE} potential was calculated by using the Nernst equation:

$$V_{RHE} = V_{Ag/AgCl} + 0.059 * pH + V^0_{Ag/AgCl} ,$$

where V_{RHE} is the converted potential *vs* RHE, $V_{Ag/AgCl}$ is the experimental potential measured against the Ag/AgCl reference electrode, and $V^0_{Ag/AgCl}$ is the standard potential of Ag/AgCl at 25° C (0.198 V).

The incident photon to current conversion efficiency (IPCE) was measured in the two-electrode configuration at the constant bias of 1V *vs* Pt electrode from 300 to 650 nm by using a tunable light source provided by a stabilized 500 W Xenon lamp combined with a computer-controlled double grating monochromator. The whole system was purchased from JASCO Co.

The oxygen and hydrogen evolution were directly measured in an airtight 2-electrodes PEC cell connected to a gas micro-chromatograph (Inficon 3000, EZ IQ). Prior to measurements, the PEC cell was evacuated and filled with Ar to atmospheric pressure repeatedly to eliminate air in the cell. The photoelectrode was biased at 1V *vs* the Pt counter electrode in pH 7 potassium phosphate buffer solution and illuminated by a simulated AM1.5 solar light. The gas probes were taken every 10 minutes by the gas micro-chromatograph.

Physical characterization. X-Ray Diffraction (XRD) measurements were performed by using Rigaku RINT-2500 XRD analyzer. The morphology of the samples was observed in Scanning Electron Microscope (SEM) JEOL JSM-7001J equipped with the EDS probe, which was used to analyze the elemental composition profiles. Scanning Probe Microscopy (SPM) analyses were performed in a semi-contact mode with a silicon cantilever for topography measurements. In order to spread resistance measurements, the ITO film underlying the nanorods was electrically biased at -2.0 V, whereas the conductive cantilevers (BudgetSewnsors, ContE-G, Cr/Pt, spring constant k=0.22 N/m, tip radius 25 nm or Multi75E-G, Cr/Pt, k=2.4 N/m, tip radius 25 nm) were grounded.

Supplementary Figures

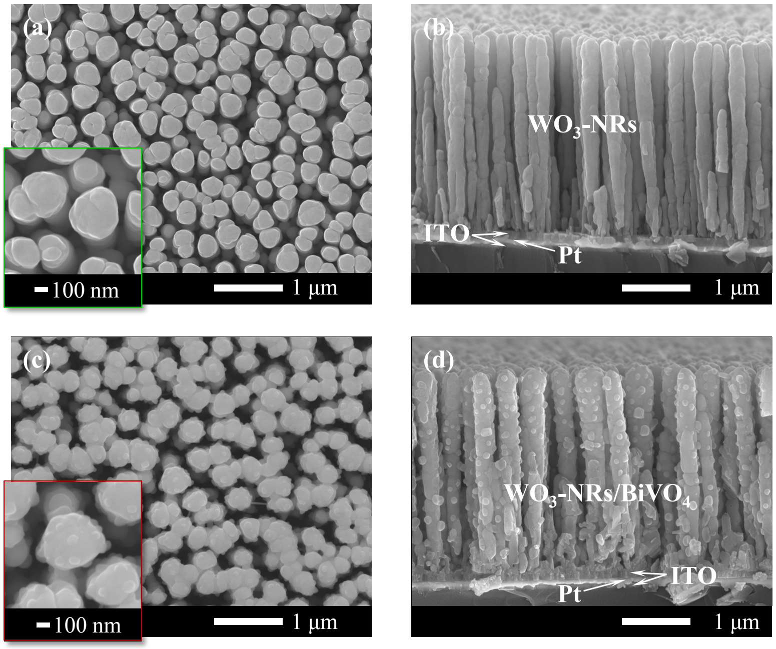

Supplementary Figure S1. Top and cross section SEM images of optimized WO_3-NRs (a, b) and core-shell WO_3-NRs/$BiVO_4$ nanostructures (c, d).

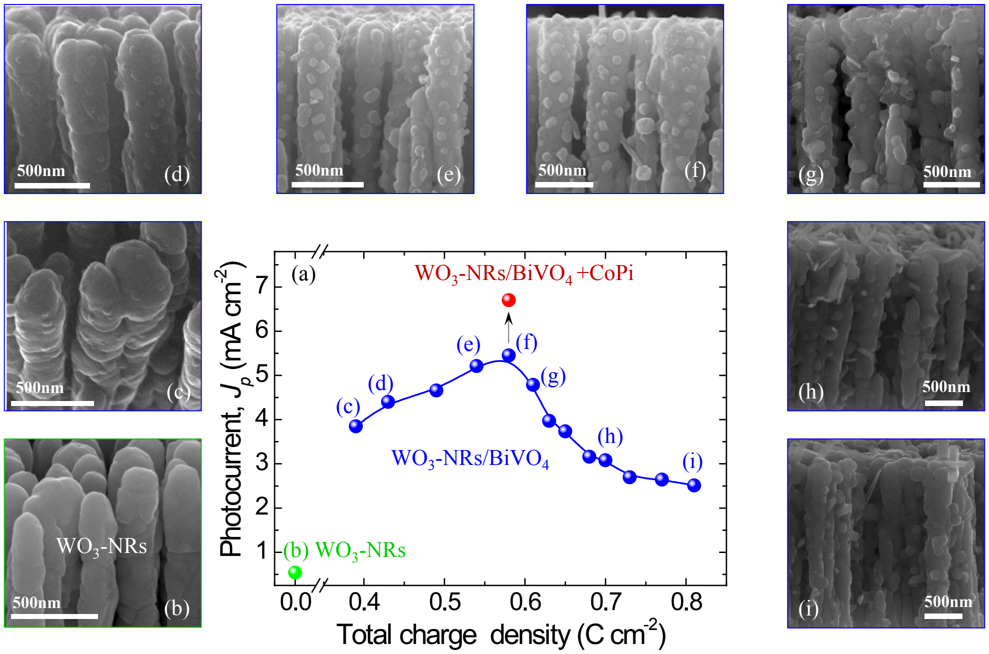

Supplementary Figure S2. Optimization of the WO_3-NRs/$BiVO_4$ photoanode. (a) Photocurrents measured at $1.23V_{RHE}$ under the standard AM1.5 illumination *vs* total charge density that passed during the electrodeposition of $BiVO_4$ for WO_3-NRs/$BiVO_4$ samples (blue) together with photocurrent values for initial WO_3-NRs (green) and optimized WO_3-NRs/$BiVO_4$+CoPi (red) samples. SEM images of initial WO_3-NRs prepared by GLAD (b) and after electrodeposition of $BiVO_4$ (c-i).

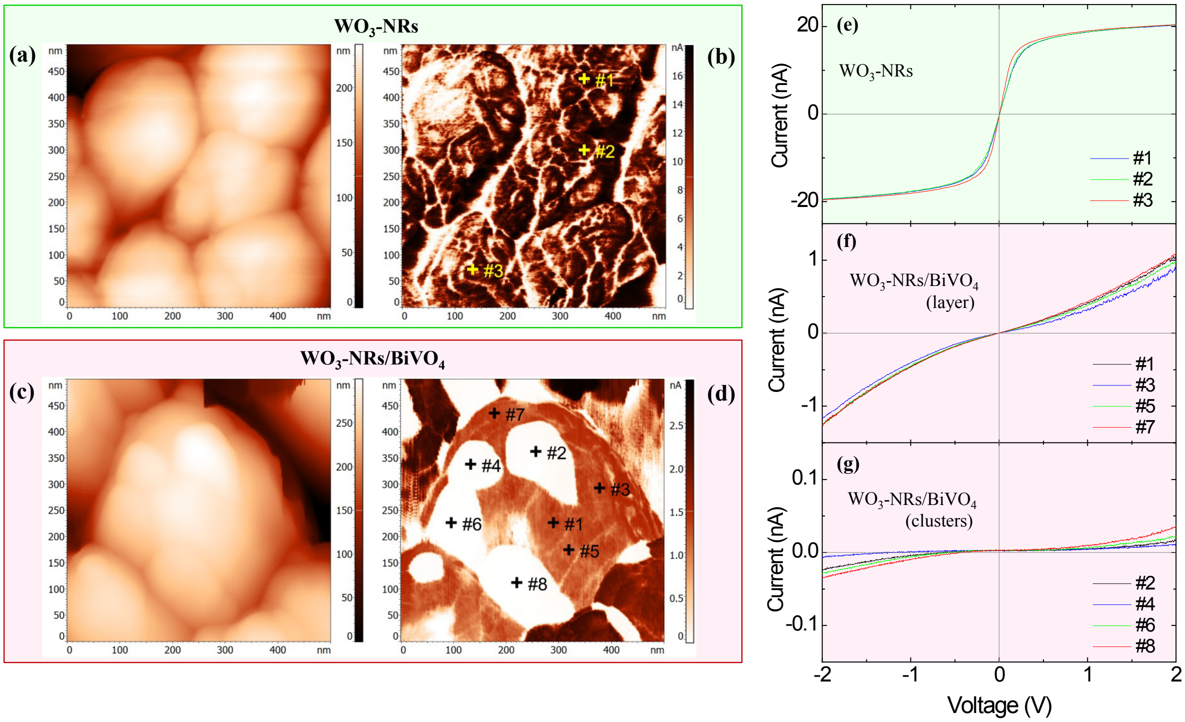

Supplementary Figure S3. SPM characterization of WO_3-NRs and WO_3-NRs/$BiVO_4$ samples: (a, c) topographies, (b, d) local current maps and (c, f, g) I-V characteristics measured at selected points.

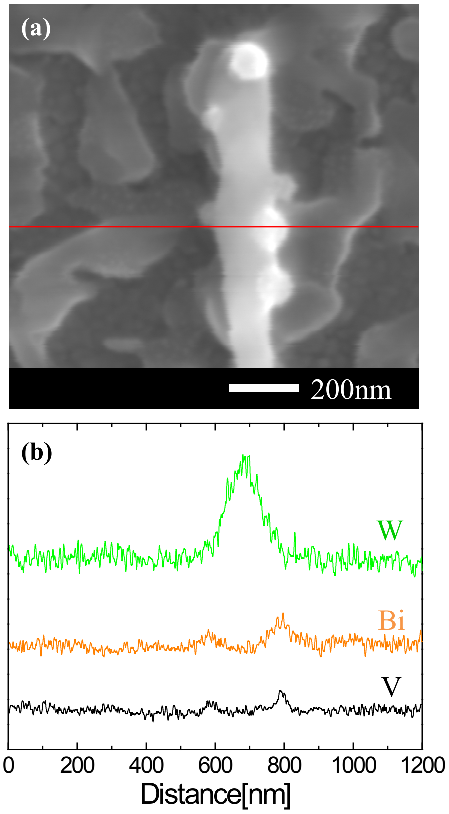

Supplementary Figure S4. (a) SEM image of a single WO_3 nanorod with a $BiVO_4$ conformal layer and (b) W, Bi and V elemental distributions across the nanorod measured by EDS.

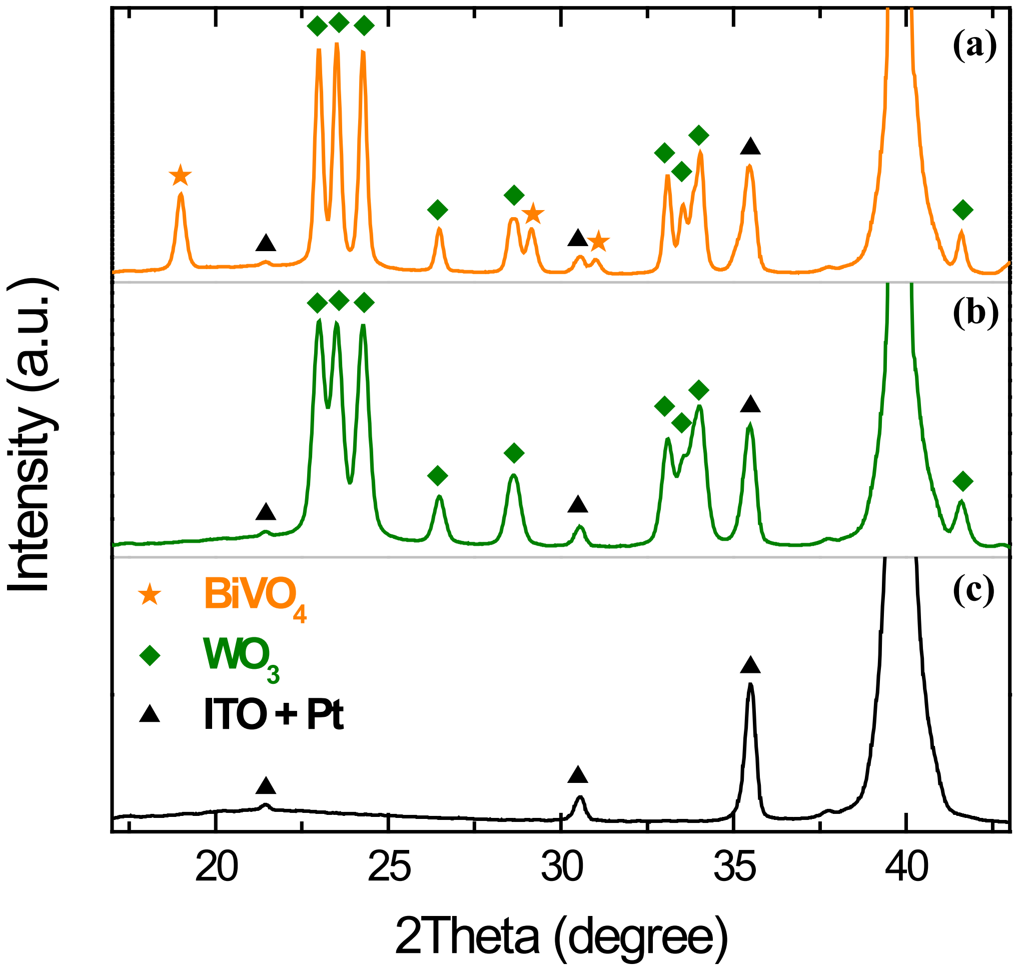

Supplementary Figure S5. XRD spectra of (a) WO_3-NRs/$BiVO_4$, (b) WO_3-NRs, and (c) ITO/Pt/ITO samples.

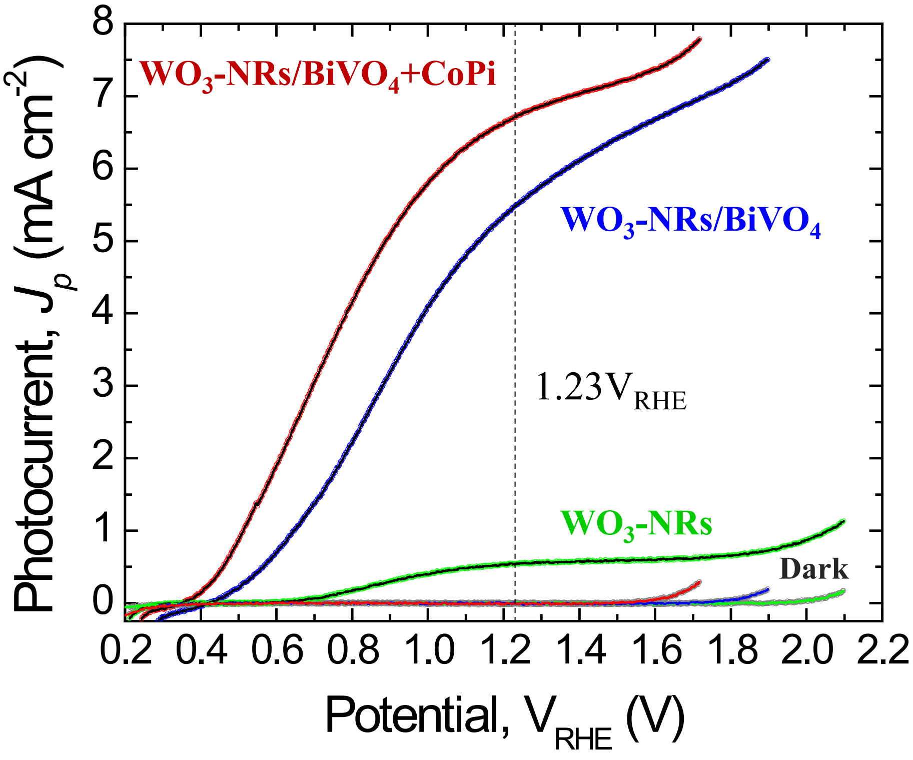

Supplementary Figure S6. I-V characteristics of the optimized WO_3-NRs (green), WO_3-NRs/$BiVO_4$ (blue) and WO_3-NRs/$BiVO_4$+CoPi (red) samples measured in a three-electrode configuration.

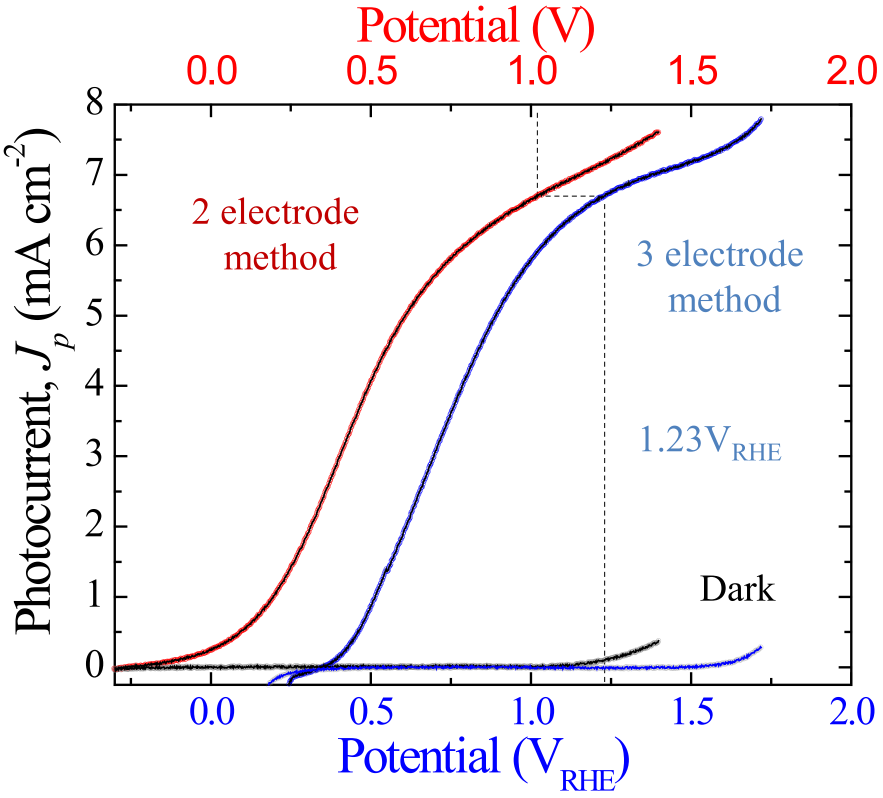

Supplementary Figure S7. Comparison of I-V characteristics of the same optimized WO_3-NRs/$BiVO_4$+CoPi photoanode measured by two-electrode (red) and three-electrode (blue) methods.

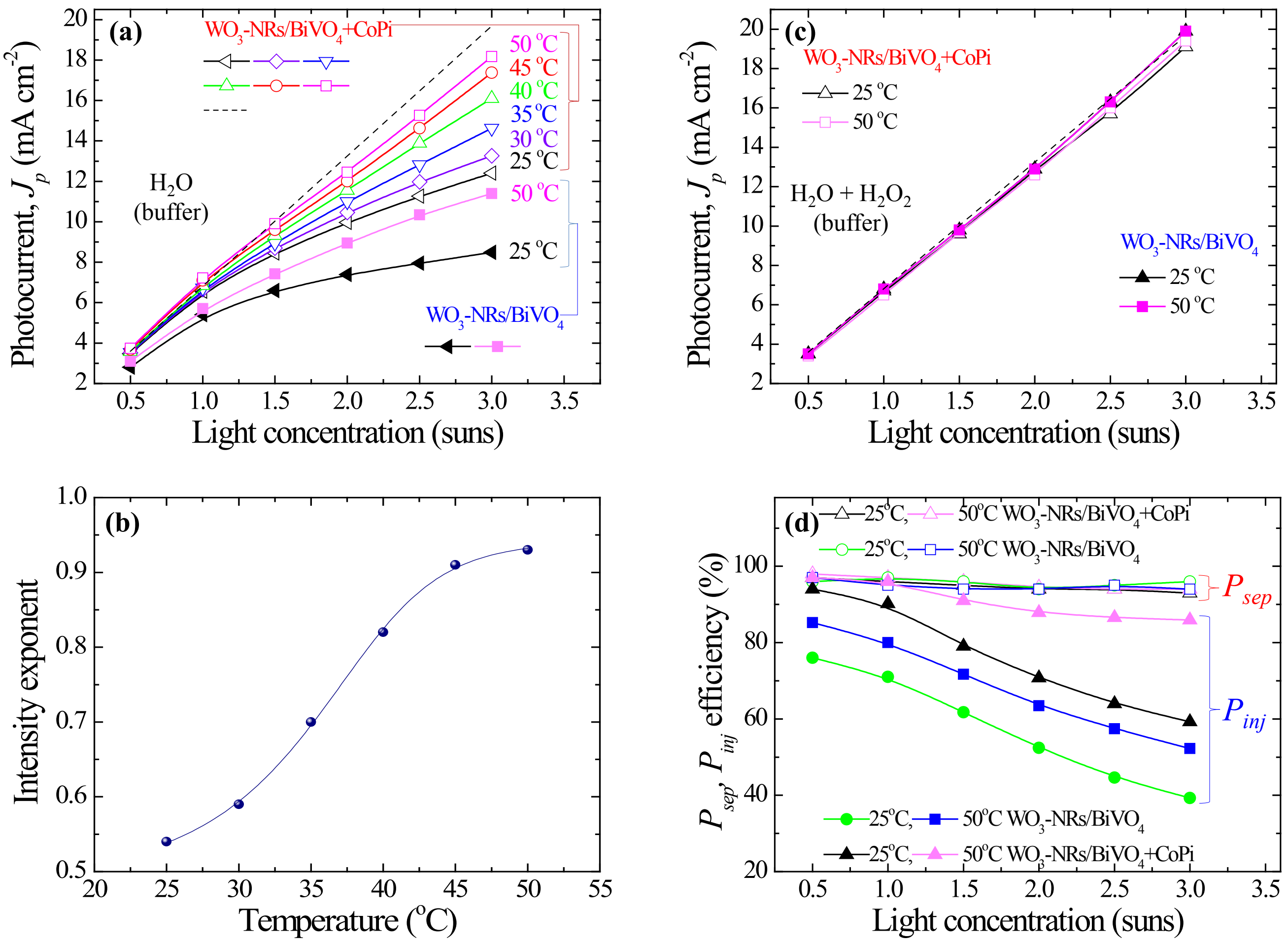

Supplementary Figure S8. Combined effect of light concentration and elevated temperature on the performance of an optimized WO_3-NRs/$BiVO_4$ and WO_3-NRs/$BiVO_4$+CoPi photoanodes. (a) Dependence of the photocurrent on light intensity measured at different temperatures (25 – 50 °C) under 1V bias *vs* Pt counter electrode. The dashed line shows a theoretical photocurrent of 6.7 [mA]$\times I^m$ [suns] with the intensity exponent $m = 1$, i.e. without additional recombination losses. (b) The intensity exponent m calculated from experimental data for WO_3-NRs/$BiVO_4$+CoPi photoanode by a linear fit of log-log plot of the photocurrent *vs* light intensity, $Lg(J_p) \sim m \times Lg(I)$. (c) Dependence of the photocurrent on light intensity at different temperatures (25 – 50 °C) under 1V bias *vs* Pt counter electrode measured in the electrolyte containing 0.5M H_2O_2 as the hole scavenger. (d) Separation (P_{sep}) and injection (P_{inj}) efficiencies vs light intensity for different temperatures calculated for the potential 1V *vs* Pt counter electrode. The absorption efficiency was assumed to be 100% with the absorption photocurrent $J_A = 7.5$ mA cm^{-2}.

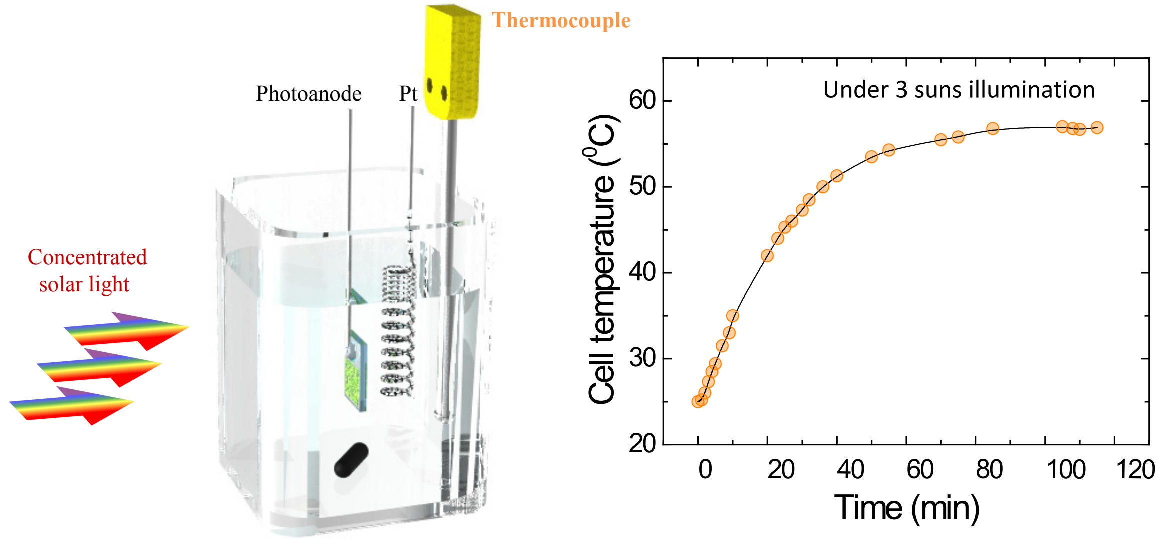

Supplementary Figure S9. Demonstration of the PEC cell heating to 50-60 °C by concentrated solar light.

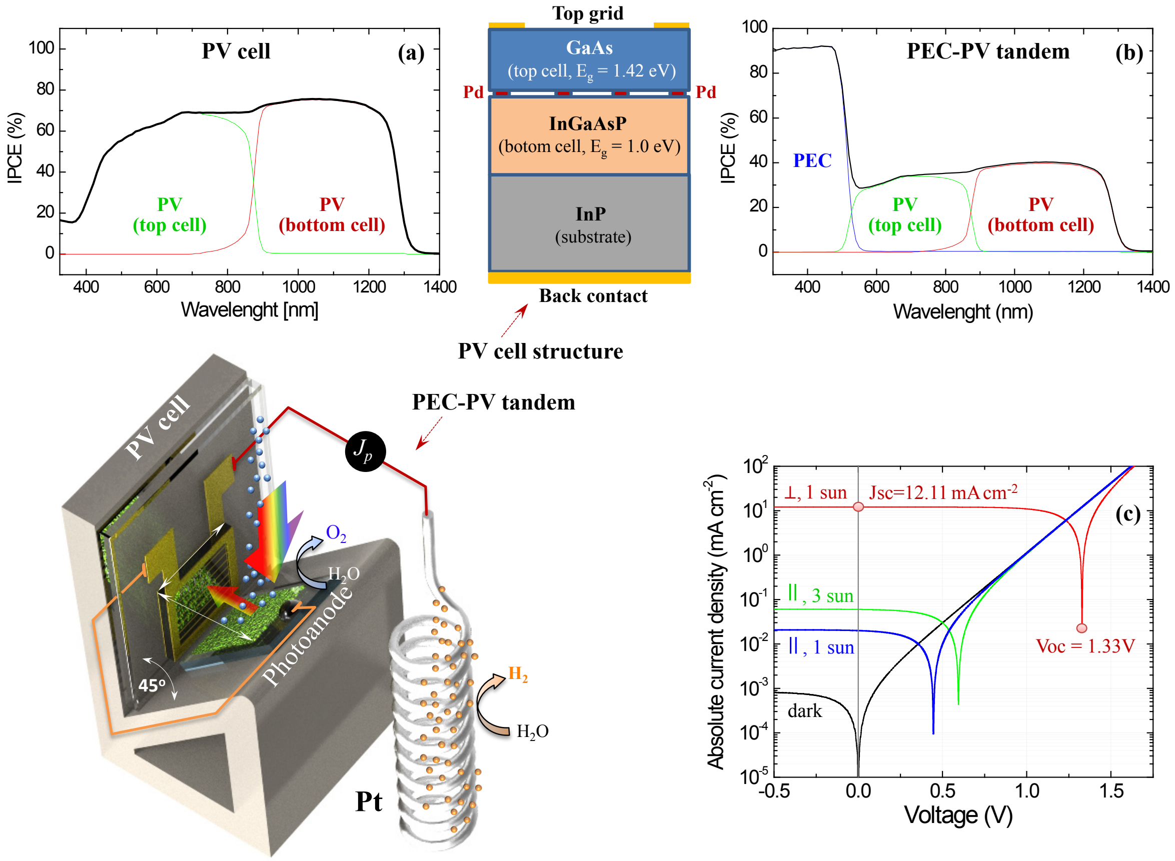

Supplementary Figure S10. Schematic illustration of the GaAs/InGaAsP double-junction PV cell structure and assembly of the PEC-PV tandem cell. (a) and (b) show IPCE spectra of the PV cell and the PEC-PV tandem, respectively. (c) I-V characteristics of the PV cell measured under normal incident light at 1 sun (red), and in parallel to the incident light in the tandem assembly without the photoanode at 1 sun (blue), at 3 suns (green) and at dark conditions (black).

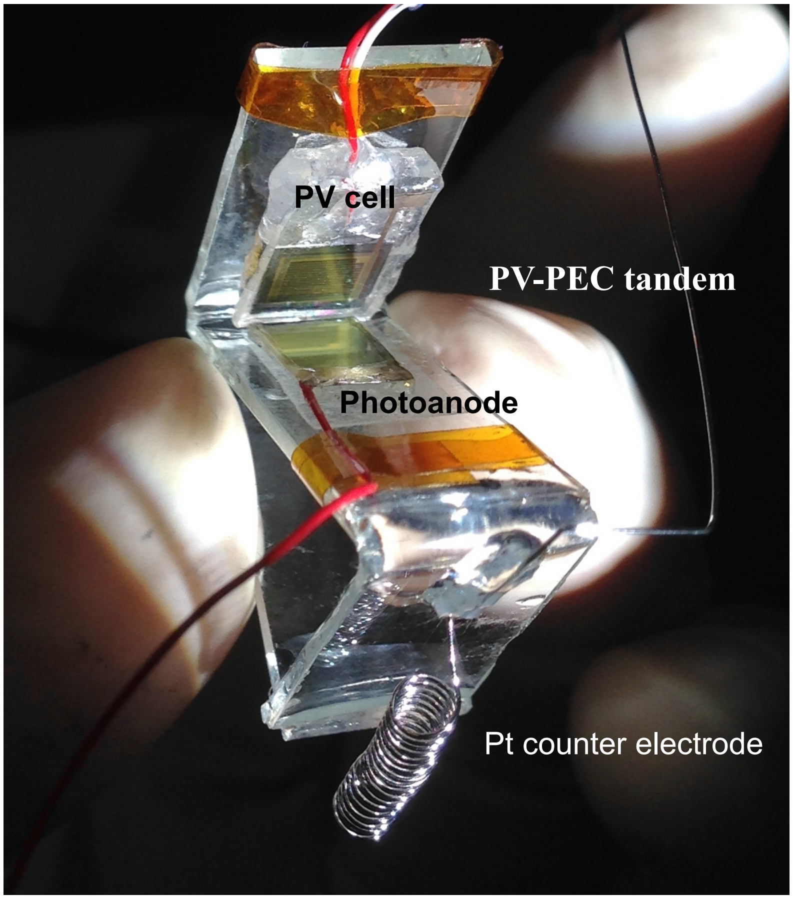

Supplementary Figure S11. Photograph of the assembled PEC-PV tandem cell.

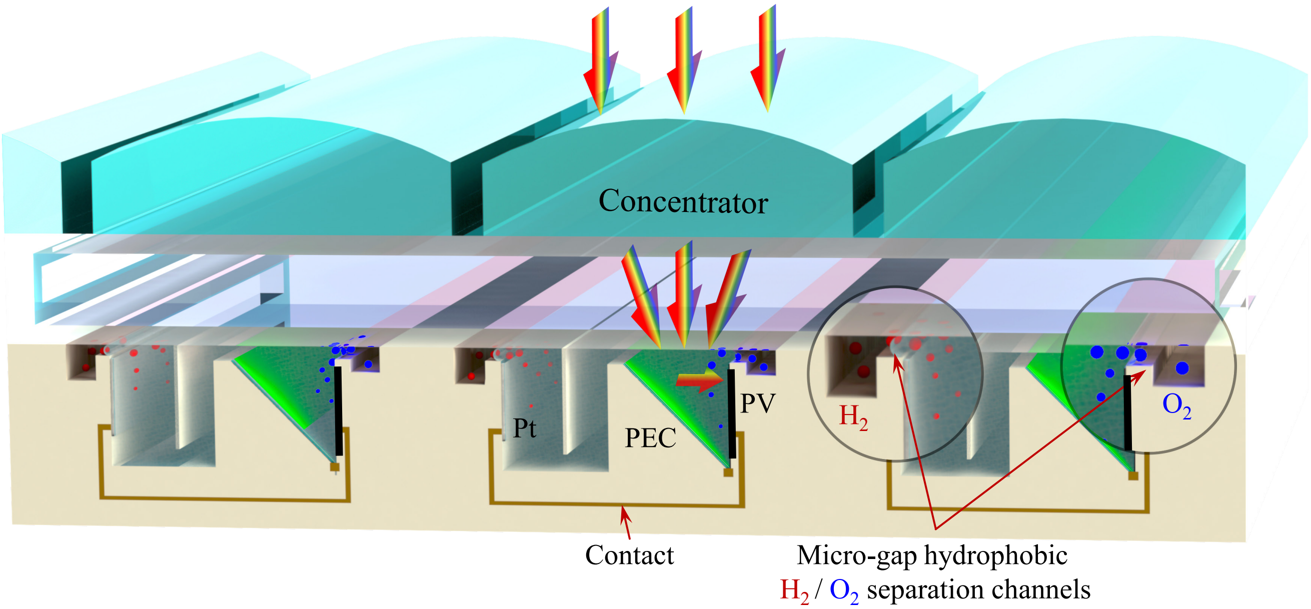

Supplementary Figure S12. Schematic illustration of a possible water splitting panel with O_2 and H_2 collecting tranches that are fitted with PEC-PV tandems, Pt counter electrodes and hemispherical light concentrators on the top of the panel module.

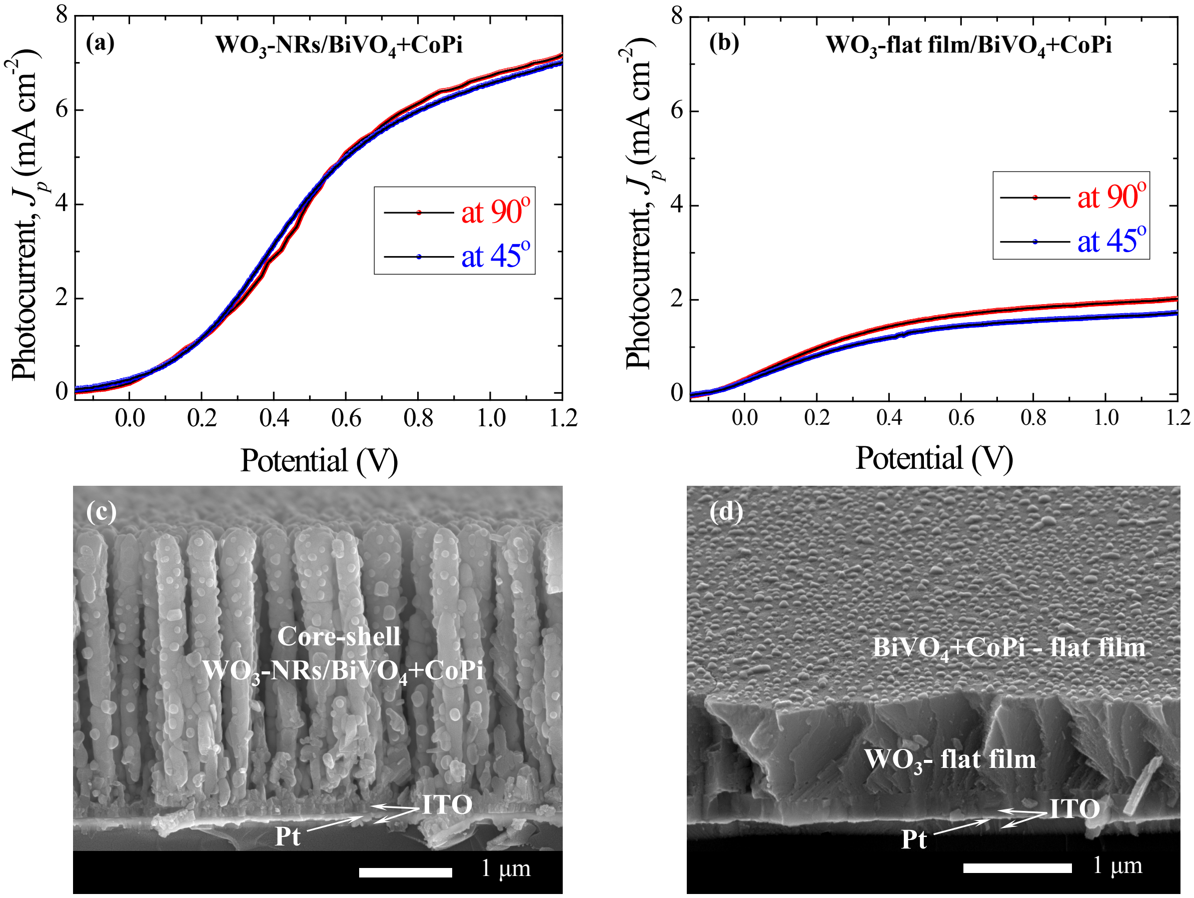

Supplementary Figure S13. I-V characteristics of the photoanodes based on core-shell WO_3-NRs/$BiVO_4$+CoPi nanostructures (a) and WO_3/$BiVO_4$+CoPi flat films (b) measured at 90° and 45° with respect to the incident light. (c,d) Cross section SEM images of the core-shell and flat film photoanodes, respectively.

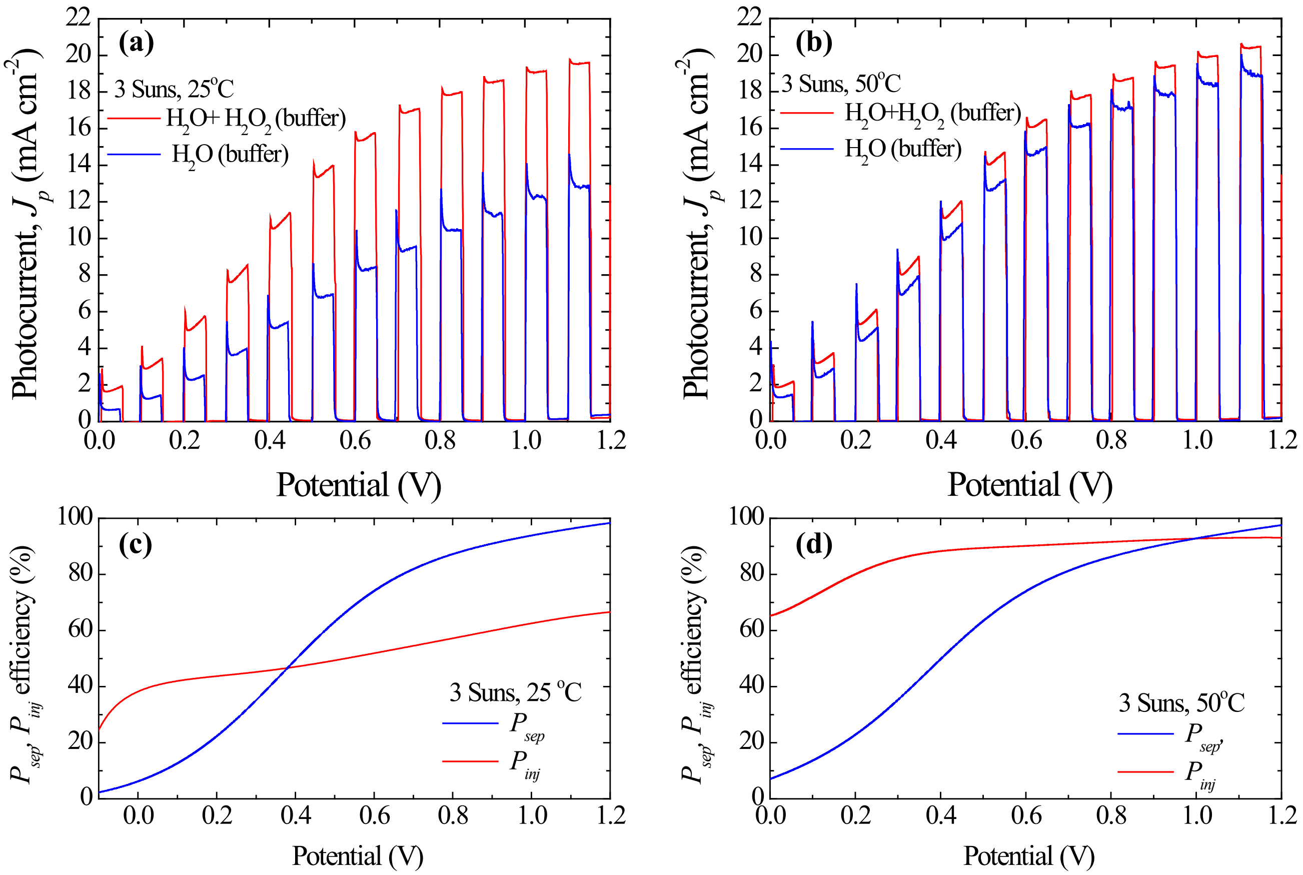

Supplementary Figure S14. I-V characteristics of the photoanodes based on core-shell WO_3-NRs/$BiVO_4$+CoPi nanostructures measured under concentrated light of 3 suns in the standard electrolyte and with hole scavenger 0.5M H_2O_2, and calculated P_{sep} and P_{inj} efficiencies at 25 °C (a,c) and 50 °C (b,d), respectively. The absorption efficiency was assumed to be 100% with the absorption photocurrent J_A = 7.5 mA cm^{-2}.
